# Esophageal Pemphigus Vulgaris: A Rare Etiology of Upper Gastrointestinal Hemorrhage

**DOI:** 10.1155/2021/5555961

**Published:** 2021-03-18

**Authors:** Jennifer Rose F. Del Castillo, Muhammad Nadeem Yousaf, Fizah S. Chaudhary, Nahar Saleh, Lawrence Mills

**Affiliations:** ^1^Department of Medicine, MedStar Union Memorial Hospital, Baltimore, MD 21218, USA; ^2^MedStar Good Samaritan Hospital, Baltimore, MD 21239, USA; ^3^MedStar Franklin Square Medical Center, Baltimore, MD 21237, USA; ^4^MedStar Harbor Hospital, Baltimore, MD 21225, USA; ^5^Department of Gastroenterology, MedStar Good Samaritan Hospital, Baltimore, MD 21239, USA

## Abstract

Pemphigus vulgaris (PV) is an autoimmune blistering disorder of the skin and mucosal surfaces characterized by acantholysis (loss of adhesion between epidermal cells). Esophageal involvement of PV is an underdiagnosed entity as routine diagnostic endoscopy is not recommended in asymptomatic patients. Dysphagia and odynophagia are common presenting symptoms; however, upper gastrointestinal bleeding (UGIB) associated with esophageal involvement of PV without a history of mucosal blistering is extremely uncommon. We present a case of esophageal involvement of PV associated with active UGIB that was diagnosed on endoscopic evaluation. This case illustrated the importance of early endoscopy to identify the esophageal involvement of PV especially in patients with preexisting disease who present with gastrointestinal symptoms such as dysphagia, odynophagia, and hematemesis. Early recognition of esophageal involvement of PV and initiation of corticosteroid and/or immunosuppressant therapy may improve the outcome of the disease.

## 1. Introduction

Pemphigus vulgaris (PV) is an autoimmune disorder characterized by intraepithelial blistering of the skin and mucous membranes due to immunoglobulin G (IgG) antibodies that attack against keratinocytes. This results in loss of adhesion between cell surfaces. The primary site of pathologic involvement is the Malpighi spinous layer, which is the deeper, nonkeratinized layer of the epidermis. Autoantibodies, specifically IgG1 and IgG4, target glycoproteins desmogleins 1 and 3 that are located in desmosomes of the skin and mucosal epithelium. Autoantibodies attack and promote the rupture of intercellular bridges consequentially forming intraepithelial clefts and thereby acantholysis. Certain genetic factors such as having the expression of histocompatibility antigen (HLA) A26 were thought to predispose an individual to develop PV. Furthermore, the presence of endogenous factors such as immunologic and exogenous causes such as viruses, drugs, and physical agents contribute in the development of PV [1].

Esophageal involvement of PV is uncommon and may be overlooked, as routine endoscopy is not performed in patients with cutaneous manifestations of PV. Dysphagia and odynophagia are common symptoms; however, patients with preexisting PV with or without prior mucosal involvement may present with upper gastrointestinal bleeding (UGIB) due to involvement of esophageal mucosa [1, 2]. Here, we present a rare case of pemphigus vulgaris without a history of oral cavity blistering lesions that present with acute UGIB of bright red blood from esophageal involvement of PV.

## 2. Case Description

**Figure 1:**
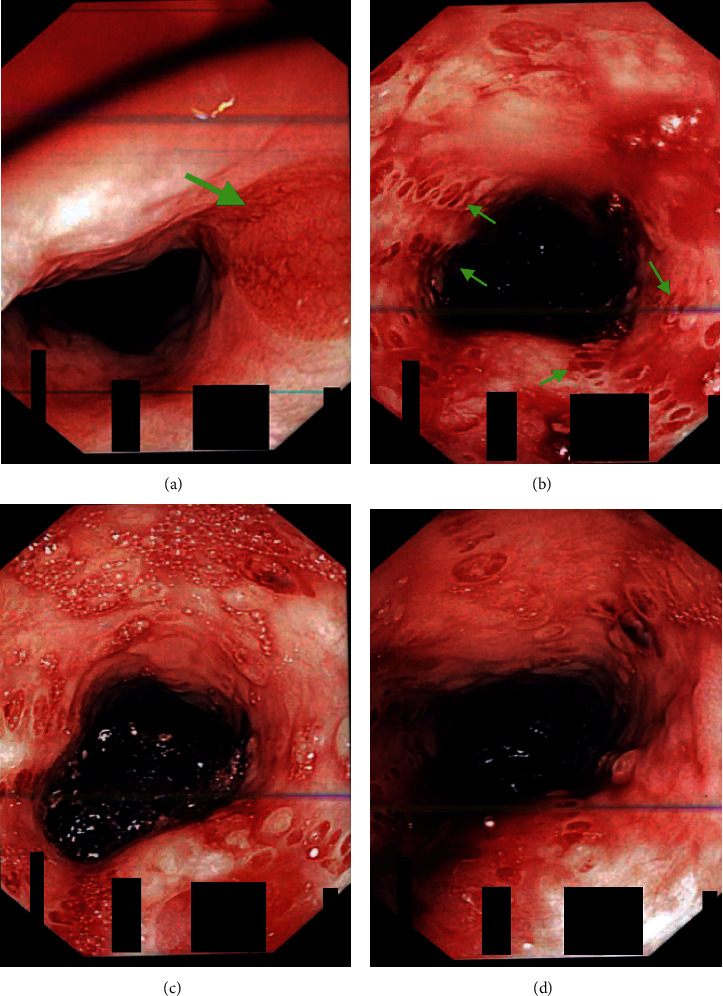
Upper endoscopic evaluation showing multiple mucosal ulcers with blisters throughout the esophagus suggestive of pemphigus vulgaris.

An 85-year-old female with a history of cutaneous pemphigus vulgaris, chronic anemia, and chronic kidney disease (CKD) stage III presented with the eruption of grouped blisters over her upper and lower extremities for 2 weeks. At presentation, she was afebrile and hemodynamically stable. On examination, multiple grouped vesicles and flaccid blisters filled with serous fluid were noted on extensor surfaces of both arms and thighs without evidence of mucosal lesions. She was also found to have an abscess on the dorsal aspect of the left foot. She was treated with IV antibiotics after incision and drainage of the abscess. Her hospital course was complicated with non-ST-elevation myocardial infarction (NSTEMI) requiring heparin infusion, followed by hematemesis of bright red blood. Heparin infusion was stopped, and esophagogastroduodenoscopy (EGD) was performed that revealed large amount of blood and clots in the pharynx to overlie the larynx and throughout the esophagus. Endoscope was withdrawn, and patient was intubated for airway protection before performing complete upper endoscopic evaluation. Multiple shallow ulcers and grouped blisters were noted throughout the proximal and midesophageal mucosa indicative of pemphigus lesions (Figure 1). Diffuse erythema on the gastric antrum was found indicative of gastritis. Unfortunately, due to the profound bleeding, biopsy of lesions was not performed. She was started on intravenous (IV) methylprednisone (40 mg) and pantoprazole (40 mg) twice daily. Her diet was gradually advanced, and methyl prednisone was switched to oral prednisone 40 mg. A complete resolution of patient's symptoms was noted on 2 weeks follow-up. Repeat EGD (2 weeks later) revealed marked improvement of bullous lesions without evidence of active bleeding or gastritis. Patient's symptoms improved without further episodes of UGIB.

## 3. Discussion

The reported incidence of PV is 0.5–3.2 cases per 100000 with majority of patients diagnosed in the fifth decade of life [1, 3]. The prevalence of esophageal involvement of PV is unknown, as they are often unrecognized until patients present with gastrointestinal symptoms and/or undergo endoscopic evaluation for other reasons. Classic presentation of PV is characterized by involvement of the skin and mucosal surface; however, oral lesions are considered the hallmark of disease in 50% of cases [4]. In an observational study of 42 patients with vesiculobullous dermatosis, EGD evaluation revealed significant involvement of upper gastrointestinal track mucosa such as oral lesions in 87% cases, while esophageal (67%), gastric (52%), and duodenal mucosal (20%) lesions were also uncommon [5]. Esophageal involvement of PV associated with UGIB in the absence of oral mucosal lesions is an extremely rare entity that was seen in our case with only 5 reported studies in the current literature (Table 1) [4, 6–9].

Patients with esophageal involvement of PV present with dysphagia (57.1%), odynophagia (21.4%), and rarely hematemesis (3.5%), though majority of individuals are asymptomatic [1, 10–12]. Upper endoscopic evaluation recognizes the esophageal mucosal lesions under direct visualization and enables simultaneous biopsy sampling for definitive diagnosis. On EGD, diffuse exfoliation of mucosal surface, multiple linear ulceration, and erosions with or without mucosal erythema are classic findings of esophageal PV that may extend from oropharynx to the lower esophageal sphincter. In cases of sever ulceration, active hemorrhage from friable mucosa are seen commonly from surface contact with advancement of the endoscope [13]. Histological examination of mucosal biopsy is diagnostic and characterized by acantholysis, clefts in the suprabasilar layer, intraepidermal vesicles, and tombstone appearance of cells [13, 14]. The diagnostic yield of indirect immunofluorescence is 75%; however, direct immunofluorescence and immunohistochemistry confirm the diagnosis of PV by identifying intracellular deposits of IgG antibodies at the site of acantholysis [13]. In our case, UGIB resulted from severe lineal ulceration through exfoliated mucosa and blistering that was further triggered with the recent use of heparin for NSTEMI management. Patient with pemphigus vulgaris complaining of any esophageal symptoms during antithrombotic therapy has a high risk of esophageal bleeding and requires caution. Bleeding may occur when anticoagulant therapy is given in PV cases with esophageal mucosal lesions. The diagnosis of PV was based on direct endoscopic evaluation of esophageal lesions. Upper endoscopy evaluation is not required in all cases of PV; however, it is reasonable to pursue in patients with hematemesis and in those with active eruption of dermoepidermal or mucosal lesions of PV prior with a medical history of bullous lesions of esophageal mucosa. In our case, endoscopy was necessary because of hematemesis. Biopsy of friable esophageal mucosa was not performed in the present case because of ongoing active bleeding.

The first line therapy for the management of PV is corticosteroids. Patients with suboptimal response to the corticosteroids are treated with immunosuppressant agents such as cyclophosphamide, mycophenolate mofetil, IVIG, or plasma exchange [15]. Ultimately, the goal is to induce remission and reduce the steroid dose to avoid complications and side effects. In this patient, high dose of IV methylprednisolone was initiated to induce clinical remission, presumably due to the severity of the clinical presentation and endoscopic findings. She was also started on proton pump inhibitor therapy for stress ulcer prophylaxis. A few days later, the patient's symptoms improved, and she was transitioned to the oral tapering dose of prednisone. Repeat endoscopy revealed improvement of the lesions. PV is the most common yet life-threatening subtype of pemphigus with a mortality rate of approximately 5–15% [3, 16]. Morbidity and mortality depend upon several factors including severity of disease, poor response to the maximum dose of corticosteroids, and presence of other comorbidities. Routine endoscopic screening for esophageal PV is not recommended in the current guidelines. However, endoscopic screening should be tailored on case-to-case basis depending upon clinical presentation of the case, high risk stigmata for UGIB and those at risk of recurrent bleeding due to extensive involvement of esophageal PV.

## 4. Conclusions

Esophageal involvement of PV is an underdiagnosed entity; however, a high index of clinical suspicion is required for early identification of esophageal lesion. Endoscopic evaluation is reasonable in patients presenting with simultaneous active skin eruption of PV with gastrointestinal symptoms such as dysphagia, odynophagia, or hematemesis. Early endoscopic identification of esophageal PV and timely treatment with corticosteroids may result in complete resolution of lesions and prevent life-threatening UGIB.

## Figures and Tables

**Table 1:** Studies with esophageal involvement of pemphigus vulgaris associated with upper gastrointestinal bleeding.

Study	Year	Age (year)/sex	Presentation	Preexisting PV	Endoscopic findings	DIF	IIF	Treatment
Wood et al. [6]	1982	56/F	Hematemesis	Yes, both cutaneous and mucosal	Multiple circumferential erythematous and granularity of lower end of the esophagus and area of discrete ulceration above the EGJ	Positive	Negative	Sucralfate and antacid
Venkataram et al. [7]	2001	26/F	Hematemesis and melena	No	Friable mucosa with erythema and linearmucosal ulcers	NA	Negative	Prednisolone, cyclophosphamide, and antacids
Tageja et al. [8]	2010	69/F	Hematemesis, peptic ulcer disease	No	Multiple hemorrhagic bullae in the midesophagus	NA	NA	Prednisone
Mohan et al. [9]	2013	48/F	Hematemesis	Yes, both cutaneous and mucosal lesions	Oropharyngeal ulcers and mucosal desquamation	NA	NA	Steroids and PPI
Chang et al. [2]	2014	41/F 30/F	Hematemesis	Yes, cutaneous PV	Mucosal edema and erythema on the esophagus and larynx with desquamation. Upper esophageal sphincter to lower esophagus with diffuse exfoliation of mucosa with multiple linear ulcer and erosions.	NA	NA	IV hydrocortisone and mycophenolate mofetil
Del castillo et al. (present case)	2020	85/F	Hematemesis	Yes	Multiple shallow ulcers and grouped blisters throughout the proximal and midesophageal mucosa.	NA	NA	Systemic steroids and proton pump inhibitor

PV, pemphigus vulgaris; DIF, direct immunofluorescence; IIF, indirect immunofluorescence; NA, not available; PPI, proton pump inhibitors.
