# Erratum to “Esophageal Pemphigus Vulgaris: A Rare Etiology of Upper Gastrointestinal Hemorrhage”

**DOI:** 10.1155/2021/9871312

**Published:** 2021-05-26

**Authors:** Jennifer Rose F. Del Castillo, Muhammad Nadeem Yousaf, Fizah S. Chaudhary, Nahar Saleh, Lawrence Mills

**Affiliations:** ^1^Department of Medicine, MedStar Union Memorial Hospital, Baltimore, MD 21218, USA; ^2^MedStar Good Samaritan Hospital, Baltimore, MD 21239, USA; ^3^MedStar Franklin Square Medical Center, Baltimore, MD 21237, USA; ^4^MedStar Harbor Hospital, Baltimore, MD 21225, USA; ^5^Department of Gastroenterology, MedStar Good Samaritan Hospital, Baltimore, MD 21239, USA

In the article titled “Esophageal Pemphigus Vulgaris: A Rare Etiology of Upper Gastrointestinal Hemorrhage” [1], the published version of Figure 1 should be rotated in clockwise direction. This also includes the arrangement of the photos to indicate location within the GI tract. This mistake was introduced during the production of the article by the publisher, and we sincerely apologise for this. The corrected figure is shown below and is listed as Figure 1.

## Figures and Tables

**Figure 1:**
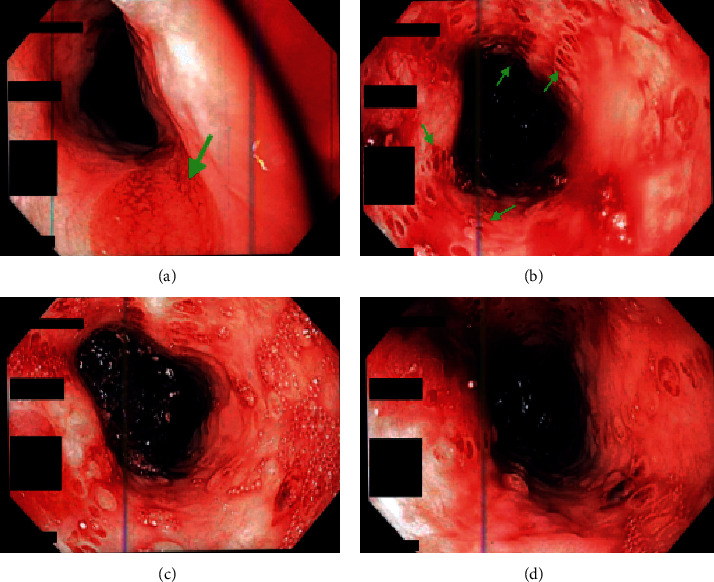
Upper endoscopic evaluation showing multiple mucosal ulcers with blisters throughout the esophagus suggestive of pemphigus vulgaris.
